# Impact of Stepping Stones on incidence of HIV and HSV-2 and sexual behaviour in rural South Africa: cluster randomised controlled trial

**DOI:** 10.1136/bmj.a506

**Published:** 2008-08-07

**Authors:** Rachel Jewkes, M Nduna, J Levin, N Jama, K Dunkle, A Puren, N Duvvury

**Affiliations:** 1 Gender and Health Research Unit, Medical Research Council, Private Bag X385, Pretoria 0001, South Africa; 2 School of Public Health, University of the Witwatersrand, South Africa; 3 Department of Psychology, University of the Witwatersrand, South Africa; 4 Biostatistics Unit, Medical Research Council, Pretoria, South Africa; 5 Behavioral Sciences and Health Education, Emory University, Atlanta, GA, USA; 6 Virology, National Institute for Communicable Disease, Sandringham, South Africa; 7 Gender Violence and Rights Team, International Centre for Research on Women, Washington DC, USA

## Abstract

**Objective** To assess the impact of Stepping Stones, a HIV prevention programme, on incidence of HIV and herpes simplex type 2 (HSV-2) and sexual behaviour.

**Design** Cluster randomised controlled trial.

**Setting** 70 villages (clusters) in the Eastern Cape province of South Africa.

**Participants** 1360 men and 1416 women aged 15-26 years, who were mostly attending schools.

**Intervention** Stepping Stones, a 50 hour programme, aims to improve sexual health by using participatory learning approaches to build knowledge, risk awareness, and communication skills and to stimulate critical reflection. Villages were randomised to receive either this or a three hour intervention on HIV and safer sex. Interviewers administered questionnaires at baseline and 12 and 24 months and blood was tested for HIV and HSV-2.

**Main outcome measures** Primary outcome measure: incidence of HIV. Other outcomes: incidence of HSV-2, unwanted pregnancy, reported sexual practices, depression, and substance misuse.

**Results** There was no evidence that Stepping Stones lowered the incidence of HIV (adjusted incidence rate ratio 0.95, 95% confidence interval 0.67 to 1.35). The programme was associated with a reduction of about 33% in the incidence of HSV-2 (0.67, 0.46 to 0.97; P=0.036)—that is, Stepping Stones reduced the number of new HSV-2 infections over a two year period by 34.9 (1.6 to 68.2) per 1000 people exposed. Stepping Stones significantly improved a number of reported risk behaviours in men, with a lower proportion of men reporting perpetration of intimate partner violence across two years of follow-up and less transactional sex and problem drinking at 12 months. In women desired behaviour changes were not reported and those in the Stepping Stones programme reported more transactional sex at 12 months.

**Conclusion** Stepping Stones did not reduce incidence of HIV but had an impact on several risk factors for HIV—notably, HSV-2 and perpetration of intimate partner violence.

**Trial Registration** Clinical Trials NCT00332878.

## Introduction

Change in sexual behaviour is the cornerstone of HIV prevention, yet relatively little research and development has been invested in interventions aimed at behaviour change in any setting.^1^ School based HIV/AIDS programmes for young people in sub-Saharan Africa have generally not been rigorously evaluated but are often weakly designed, and evaluations suggest they have little impact on sexual behaviours.^2^ In this respect they are no different from programmes for adolescents in other countries that have rarely shown sustained behaviour change.^3^ Three randomised controlled trials have been conducted in Africa with both behavioural and other interventions (management of sexually transmitted infection, microfinance, community action, or health service strengthening) in community and school settings.^4^ ^5^ ^6^ Two studies showed no effectiveness in prevention of sexually transmitted infections,^5^ ^6^ but one had a positive effect on self reported behaviour.^5^ The third showed that the intervention was associated with a reduced prevalence of curable sexually transmitted infections. The incidence of herpes simplex type 2 virus (HSV-2) was lower in the group that had only a behavioural intervention but not in the group that had both this and treatment for sexually transmitted infections, and so the authors do not attribute the effect in the behavioural arm to success of their intervention.^4^ The failure to show a biological impact is a particularly important weakness as there are known limitations to the validity of self reported change in sexual behaviour, with a potential for interventions to bias reporting towards socially desirable behaviours, and because an effect on sexually transmitted infections is the ultimate objective of these interventions.^5^ ^7^ ^8^

None of the intervention programmes previously evaluated was established and widely used before the research was conducted. This is potentially an important weakness as development of interventions is an iterative process, and interventions are generally strengthened by being more extensively tested and adapted.^9^ In this respect Stepping Stones is a quite different intervention as it has been widely used for many years.^10^ It was originally developed for use in Uganda in 1995 and has been used in over 40 countries, adapted for 17 settings (including South Africa in 1998^11^), translated into 13 languages, and used with hundreds of thousands of individuals.^12^ It is almost certainly the most widely used intervention of its kind in the world. Stepping Stones is a participatory HIV prevention programme that aims to improve sexual health through building stronger, more gender equitable relationships. We conducted a trial to assess the impact of Stepping Stones on the incidence of HIV and HSV-2 and sexual practices among men and women in rural areas in the Eastern Cape province of South Africa.

## Methods

### Recruitment and randomisation

In this randomised trial we used a cluster design because the intervention is delivered to groups. The setting was historically a subsistence farming region within a radius of 1.5 hours’ drive from the town of Mthatha, where contemporary households are primarily supported by contributions from family working elsewhere, grants, and pensions. The area has two sizeable towns, seven small towns, and many villages. There are 12 hospitals, and most villages have a clinic that distributes free condoms. The unit of randomisation was a geographically defined area in which we recruited one pair of single sex groups. Details of the study design have been described previously.^13^

The 70 study clusters comprised 64 villages and six townships. Eligible locations were about 10 km from the nearest cluster (to minimise contamination of study arms), had a senior or junior secondary school, and a community willing to participate (established through a process of community mobilisation^13^). Clusters were grouped into seven strata, with one stratum comprising the townships and six having the villages grouped according to proximity to particular roads. Within each stratum, equal numbers of clusters were allocated to each arm. The study statistician (JL) based in Pretoria, who had no knowledge of the study area, randomly generated the allocation sequence for each stratum. The project manager (MN) and field work coordinators in Mthatha identified and randomised the clusters and then enrolled participants. There was no blinding and for logistical reasons randomisation was done before village recruitment.

In each cluster we recruited about 20 men and 20 women volunteers. Those eligible were aged 16-23, normally resident in the village where they were at school, and mature enough to understand the study and the consent process. There was a difference between the actual and intended age of participants, which is discussed in detail elsewhere.^13^ Most were recruited from schools. In each cluster recruitment started with general community mobilisation, and the study was explained to key local figures. In most villages the chief (or his representative) called a monthly community meeting. Typically the staff member attended and made a brief presentation and then took questions from the community, including from parents of potential participants. After the community meeting project staff went to the school to raise interest in the study and invited possible participants to a meeting. Here they explained the study to a group of about 60 young men and women in the targeted age group. Names were taken and the group was asked to decide on the 40 people who were most likely to be able to participate in the study. The presenter read aloud the study’s consent form to the 40 and gave an opportunity for questions. The form explained the procedures that would occur in some detail. After the group presentation we asked for confirmation that there was still general interest in participation and asked the young people to talk with their families before committing themselves. Each potential participant received a Xhosa language leaflet describing the study in terms understandable to a lay audience. Those who decided to participate were asked to report at an assigned time anywhere from two to seven days later. At that time, they provided and signed formal informed consent forms and study recruitment was finalised.

We used the method of Hayes and Bennett^14^ to calculate the sample size—that is, the number of clusters required in each arm. The calculation assumed that the effect (as measured by incidence rate ratio) would be homogeneous for men and women and assumed a two year cumulative HIV incidence rate (averaged over men and women) in the control arm of 12% and that two year incidence results would be obtained for at least 14 women and 14 men per cluster (thus allowing up to 30% of women and 30% of men to be either lost to follow-up or to be HIV positive at baseline). To calculate the sample size we needed an estimate of *k*, the coefficient of variation between clusters for the outcome measure; we used *k*=0.35 on the basis of an analysis of the results for the Eastern Cape of the 1999 national antenatal HIV seroprevalence survey, in which the clusters were antenatal clinics. A sample size of 35 clusters per trial arm would then give more than 80% power to detect as significant at the 5% level a 50% reduction in HIV incidence.

### Intervention and implementation

We compared the impact of the South African Stepping Stones (second edition)^15^ and the control intervention for groups of men and women on incident infections of HIV and HSV-2 and sexual behaviours. Our study was an effectiveness trial, rather than an efficacy trial, with the programme implemented as if in a broader community roll-out.^13^ The interventions were facilitated by project staff, who were employed by our partner non-governmental organisation the Planned Parenthood Association of South Africa (PPASA), and trained, supervised, and shown how to implement the programmein accordance with its practices. Facilitators were the same sex as the participants and either the same age or a little older. Most had further education or had undergone life skills training and were selected, in part, for their open mindedness and gendersensitivity. After three weeks of training and two practice groups, 11 facilitators delivered the Stepping Stones intervention. Another four, who were trained for four days, administered the control intervention. These two groups of facilitators were trained and supervised separately to reduce contamination.

Stepping Stones uses participatory learning approaches, including critical reflection, roleplay, and drama and draws the everyday reality of participants’ lives into the sessions. It is delivered to single sex groups, which are run in parallel, and has 13 three hour long sessions that are complemented by three meetings of male and female peer groups and a final community meeting. The programme spanned about 50 hours and ran for six to eight weeks. The sessions covered how we act and what shapes our actions; sex and love; conception and contraception; taking risks and sexual problems; unwanted pregnancy; sexually transmitted diseases and HIV; safer sex and condoms; gender based violence; motivations for sexual behaviour; dealing with grief and loss; and communication skills. The sessions were mainly held on school premises after school hours.

Our South African adaptation has a slightly different content from the Welbourn original and was not used in a community development context. Welbourn recommended working with older men and women in each community as well as young people and suggested that peer groups be encouraged to continue to meet after the end of the workshops.^10^ We did not implement either of these components as it would have added greatly to the cost and we wanted to test a delivery model that we thought could be more easily funded for roll-out. The control intervention was a single three hour session on HIV, safer sex, and condoms. The content was taken from Stepping Stones.

We administered questionnaires and collected blood samples before the intervention (baseline) and after about one and two years. The baseline interviews and intervention were staggered over a 12 month period (March 2003-March 2004), as was each round of follow-up. Participants were located for repeat interviews by using details collected at enrolment. If they had moved within the study area, they were interviewed in their new location or invited to come to the office. We also went to Cape Town, East London, and Gauteng province to conduct interviews with migrants. All participants were given 20 rand (about £1.30, €1.60, $2.50) after each interview.

We had an active community advisory board and data safety and monitoring board. After the group discussions, participants were asked to sign an informed consent form to participate on the day of the interview. The consent form was in two parts with consent to participate in the trial separated from consent for the blood tests. A trained nurse counsellor provided counselling before HIV testing to groups of eight to 10 people after they had enrolled in the study, signed consent for the interview, and completed the baseline questionnaire. Counselling typically involved five minutes of information provided by the nurse followed by 20 minutes of questions. Afterwards participants signed consent for the HIV test (they could raise issues privately then if they wanted) and they were asked whether they wanted to be told their results. If so, a study nurse gave them test results with counselling some weeks later. Participants could change their mind and get their results at any stage. Those with positive results were told their CD4 counts and screened for medical problems. They were also referred to local health services and HIV support groups according to a referral algorithm that took into account their clinical condition and the available community services as well as locally accepted standards of care. The Medical Research Council paid for lunch, transport, and consultation fees for HIV positive participants accessing health services. The study nurses supported participants with social problems and HIV related problems throughout the course of the study, referring them to social workers or health facilities as appropriate. During the study anti-retroviral drugs became available in the public sector and at this point the consent form was changed to ask participants who had opted not to collect their result if they would like to be told if they tested positive.

### Laboratory methods

The primary outcome measure was HIV incidence, determined through blood tests at baseline and at 12 and 24 months. All blood tests were conducted blind to the treatment arm. HIV status was assessed with two rapid tests by using the World Health Organization’s testing algorithm.^16^ We used the Determine (Abbott Diagnostics, Johannesburg) screening test and retested samples with positive results with Uni-gold (Trinity Biotech, Dublin, Ireland). We carried out an HIV-1 enzyme linked immunosorbent assay (ELISA) (Genscreen) followed by two confirmatory ELISAs (Vironostika and Murex 1.2.0) if the sample was positive for HIV to clarify any indeterminate results. Towards the end of the second round of interviews collection of dried blood spots was introduced (in 357 cases) as it was easier logistically and more acceptable for participants. In the third round of interviews most blood was collected as dried blood spots (n=1530). These were tested with a screen ELISA (Genscreen) and positive results were confirmed with a second ELISA (Vironostika). Participants were not given a choice of the method of blood collection, which was chosen purely on fieldwork logistics. There was no difference between arms in the use of dried blood spots at 24 months but there was a small difference (7%) at 12 months, with more in the control arm. The dried blood spot method has been used extensively in South African populations over the past few years but has not been specifically validated for South Africa. The HIV tests on the dried blood spots were optimised by use of paired serum samples and dried blood. The National Institute for Communicable Diseases participates in a programme supported by the Center for Communicable Diseases Control that has shown the methods used can optimally identify HIV-1 from dried blood spots.

We used two glycoprotein G based HSV-2 ELISAs to test for herpes infection, Kalon (Kalon Biological, Aldershot, UK) and HerpeSelect Immunoblot IgG (Focus Technologies, Cypress, Ca, USA). We used an additional test, CAPTIA herpes simplex virus (HSV) IgG type specific ELISAs to resolve discrepant results. The testing for HSV-2 on dried blood spots was optimised with paired serum samples and dried blood spots as described in Hofgrefe et al.^17^ The CAPTIA HSV-2 assay had not been validated for use as a confirmatory assay at the time of the study.

We assessed the impact of the intervention on behaviour and attitudes with a questionnaire administered in Xhosa. Table 1 describes the outcome measures, indicators, and assessment[Table tbl1] and further details can be found elsewhere.^13^

**Table 1 tbl1:** Outcome measures in Stepping Stones programme for HIV prevention

	Indicator	No of items*	Expected direction of change because of intervention
**Primary outcome**			
Incidence of HIV	HIV seroconversion in individuals who were HIV negative at baseline	NA	Decrease
**Other outcomes**			
Incidence of HSV-2	HSV-2 seroconversion in individuals who were HSV-2 negative at baseline	NA	Decrease
No of partners	No of main partners, one-off partners, and ongoing non-primary partners (makhwapheni) since last interview	3	Decrease
Any transactional sex with casual partner	Sex primarily motivated by material gain to female casual partner since last interview, defined as provision of food, cosmetics, clothes, transportation, items for children or family, school fees, somewhere to sleep, or cash. Giving for men and receiving for women	17	Decrease
>1 incident of physical or sexual intimate partner violence	More than one episode of physical or sexual intimate partner violence since last interview	9	Decrease
Rape or attempted rape	Rape or attempted rape of non-intimate partner since last interview	5	Decrease
Correct condom use at last sex	Use of condom at last sexual intercourse for each coital act without user error or breakage	6	Increase
Any casual partner	Any one-off partner or ongoing non-primary partner (makhwapheni) since last interview	2	Decrease
Unwanted pregnancy	Pregnancy since last interview with indication that at the time she became pregnant she wanted to become pregnant “later” or “not at all”	2	Decrease
Depression	Depression in past week measured with CES-D (cut-off point ≥21)	20	Decrease
Problem drinking	Problem drinking measured with AUDIT scale (cut-off point ≥9)	11	Decrease
Ever misused drugs	Misuse of cannabis, benzene, mandrax, injecting drugs, or other drug	5	Decrease

*No of questions used to assess each indicator. NA=not applicable.

### Data analysis

We followed an intention to treat approach, in which we included in the analysis all participants with evaluable data for the outcome measure under consideration. We stratified the analyses for incidence of HIV and HSV-2 by sex and carried out a test of homogeneity of treatment effect over the sexes. All other analyses were carried out separately for men and women. Participants were included in the analysis of the primary outcome only if they were HIV negative at baseline; those who had missing HIV results at baseline were excluded even if they had a subsequent negative result as they could not have been included if they had tested positive at the subsequent visit. For each participant we calculated the person years of exposure as the time from baseline to the last negative result if the person remained negative, or as the total time between any negative tests as well as half the time between the last negative and first positive tests. The primary analysis was carried out by fitting generalised linear mixed models (GLMMs) as advocated by Murray.^18^ The GLMM used a log link and assumed an underlying Poisson distribution and included terms for stratum, sex, age of respondent, baseline prevalence of HIV and HSV-2of the cluster for men and women, and treatment, with clusters being treated as a random effect. Homogeneity of the treatment effect over men and women was established by testing for a sex by treatment interaction. Generalised estimating equation (GEE) models were also fitted to test the robustness of the GLMMs. In addition we carried out cluster level analyses. Firstly, we calculated the cluster level incidence rate for each cluster, separately for men and women. These rates were compared between treatment arms by fitting a general linear model to the 140 cluster level rates (70 for men and 70 for women) with terms for the baseline cluster level prevalence, stratum, sex, and treatment arm. The results of fitting these models were used to estimate the number of HIV or HSV-2 infections prevented by the intervention over a two year period per 1000 participants. In addition, we fitted a cluster level Poisson model using the number of events and the total person years of exposure for each cluster, with terms for cluster prevalence at baseline (separate for men and women), stratum, sex, and treatment arm. These results were used as a check on the results obtained from the GLMMs. In all cases, apart from reporting the number of infections prevented, the results presented are those from the GLMMs.

We analysed other outcomes separately for men and women and for the 12 and 24 month visits. “Correct condom use on last sex” was analysed as a binary outcome with a GLMM with a logit link and underlying binomial distribution (that is, a random effects logistic regression model). The model contained terms for stratum, age of the respondent, and treatment arm. Any casual partner since the last visit, transactional sex with a casual partner since the last visit (giving for men and receiving for women), more than one incident of physical or sexual abuse since the last visit (perpetration for men and receipt for women), unwanted pregnancy since then, and any rape or attempted rape against a non-partner since then (men only) were also treated as binary outcomes. As the likelihood of these events happening might be higher with longer periods between interviews, we included the time between interviews as a covariate. Thus for each outcome we fitted random effects logistic regression models with terms for stratum, age of the respondent, time since the last visit, and treatment arm. The number of sexual partners since the last interview was analysed by first applying a square root transformation to this outcome as this was found to be variance stabilising and to lead to approximately normally distributed residuals in an analysis ignoring the clustering. A mixed model was then fitted to the transformed outcome with terms for stratum, age of the respondent, time since the last visit, and treatment arm. The effects were back transformed for easier interpretation. We analysed depression (CES-D scale), problem drinking (AUDIT scale), and drug misuse using models similar to those for correct condom use. In all cases GEE models were also fitted to confirm the results of the GLMMs. In the case of drug misuse at month 24 for women (that is, having started drug misuse between the 12 month and 24 month interview) the results presented are those for the GEE model as the GLMM failed to converge because of the small number of women misusing drugs. In all other cases the results reported are those from the GLMM.

## Results

The figure shows the trial profile.[Fig fig1] No clusters were lost to follow-up. Twelve month follow-up rates for women with known HIV status at baseline were 75.8% and 75.3% in the intervention and control arms and 75.1% and 71.8% for men in the intervention and control arms, respectively. At 24 months, 73.1% (intervention) and 76.0% (control) of women with baseline HIV results were retested and 69.5% (intervention) and 69.2% (control) of men were tested again for HIV. Loss to follow-up was mainly because participants had moved and could not be located. At baseline, 9.8% of men and 6.3% of women had a main partner also in the study. 

**Figure fig1:**
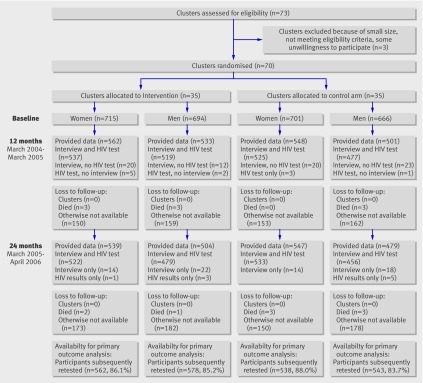
**Fig 1** Trial profile

Eighteen participants died during the main study and one committed suicide in the pilot study (figure). Causes of death in the main study were interpersonal violence (six), suicide (three), injuries from traffic incidents (two), and a range of natural causes (seven), including AIDS (one). Four of the non-natural deaths were in the control arm and seven in the intervention arm. All deaths were investigated and none was linked to activities of the study. There were no other serious adverse events.

From the available attendance registers (an incomplete set), 90 (16.8%) men and 63 (12.5%) women did not participate in any of the Stepping Stones sessions, and 189 (31.7%) men and 228 (35.7%) women did not attend the short intervention. Some 324 (60.7%) men and 298 (59.1%) women attended 75% or more of the Stepping Stones sessions, and 147 (27.5%) men and 128 (25.4%) women attended the complete programme. 

Table 2 shows the participants’ baseline characteristics. The two arms were similar for both sexes, although participants in the control arm were slightly more educated (P=0.09 for women, P=0.08 for men).

**Table 2 tbl2:** Social and demographic characteristics of two study arms. Figures are numbers (percentages)

	Women			Men	
	Intervention (n=715)	Control (n=701)		Intervention (n=694)	Control (n=666)
Age (years):					
15-17	329 (46.0)	289 (41.2)		234 (33.7)	182 (27.3)
18-19	261 (36.5)	250 (35.7)		259 (37.3)	264 (39.8)
20-21	102 (14.3)	128 (18.3)		160 (23.1)	164 (24.6)
22-26	23 (3.2)	34 (4.8)		41 (5.9)	56 (8.4)
Education grade:					
<9	67 (9.4)	40 (5.7)		112 (16.1)	79 (11.9)
9	343 (48.0)	248 (35.4)		335 (48.3)	243 (36.5)
10	243 (34.0)	279 (39.8)		188 (27.1)	229 (34.4)
11	58 (8.1)	121 (17.3)		55 (7.9)	105 (15.8)
>11	4 (0.6)	13 (1.8)		4 (0.6)	10 (1.5)
Current schooling	707 (98.9)	678 (96.7)		680 (98.0)	640 (96.2)
HIV seroprevalence	70 (9.8)	90 (12.8)		12 (1.7)	14 (2.1)
HSV-2 seroprevalence	194 (27.6)	213 (31.0)		70(10.3)	65 (10.0)
Ever had sexual intercourse	655 (91.6)	633 (90.3)		654 (94.2)	624 (93.7)
Correct use of condom on last sexual intercourse	266 (40.7)	288 (45.6)		292 (44.6)	303 (48.6)
>2 sexual partners in past year	62 (9.5)	71 (11.2)		317 (48.5)	314 (50.3)
Casual partner in past 12 months	138 (21.1)	146 (23.1)		379 (58.0)	378 (60.6)
Ever had transactional sex	177 (27.1)	141 (22.3)		200 (30.6)	168 (26.9)
More than one incident of physical/sexual intimate partner violence	177 (24.7)	157 (22.4)		100 (14.5)	96 (14.5)
Rape or attempted rape	—	—		126 (18.2)	141 (21.3)
Depression (CES-D)	117 (16.4)	103 (14.7)		45 (6.5)	54 (8.1)
Problem drinking	28 (3.9)	19 (2.7)		170 (24.5)	171 (25.7)
Ever misused drugs	37 (5.2)	53 (7.6)		256 (37.1)	262 (39.5)

Table 3 shows the results for the comparison of incidence rates of HIV and HSV-2 between the two study arms. After adjustment for stratum, baseline HIV prevalence in the cluster, and age of the respondent, Stepping Stones had little effect on the incidence of HIV. The incidence of HSV-2 was significantly lower in the Stepping Stones arm than the control arm (incidence rate ratio 0.67, 95% confidence interval 0.46 to 0.97, P=0.036). This represents a 33% reduction in incidence and translates to 34.9 (1.6 to 68.2) infections being prevented over a two year period per 1000 people in the programme. There was no evidence of heterogeneity—that is, the effect of Stepping Stones on incidence of HSV-2 was similar for men and women.

**Table 3 tbl3:** Incidence of HIV and HSV-2 according to intervention

	Stepping Stones			Control		P value for homogeneity	Adjusted* incidence rate ratio (95% CI)	P value	Coefficient of variation
	No of events	Rate/100 person years		No of events	Rate/100 person years				
**HIV**									
Overall	72	3.46		81	4.07	0.56	0.95 (0.67 to 1.35)	0.78	1.02
Women	57	5.65		68	6.95				0.81
Men	15	1.40		13	1.29				1.60
**HSV-2**									
Overall	57	3.24		75	4.62	0.91	0.67 (0.47 to 0.97)	0.036	1.13
Women	43	5.35		57	7.71				0.93
Men	14	1.46		18	2.04				1.58

*Adjusted for stratum, sex, participant’s age, and baseline cluster prevalence of HIV or HSV-2, respectively.

Table 4 shows the results of the analysis of the other outcomes for women. There was no evidence of difference in the expected direction between the two arms in any of these outcomes. At 12 months the proportion of women who had transactional sex with a casual partner since the first interview was higher in the Stepping Stones arm. It is worth noting, however, that there was little difference between the two arms in the proportions of women who had a casual partner and that the difference in the proportions having transactional sex with a casual partner had disappeared by month 24. There was slight evidence (P=0.11) that the incidence of pregnancy was higher in the Stepping Stones arm at 24 months.

**Table 4 tbl4:** Other outcomes at 12 and 24 months in women according to intervention

	Stepping Stones			Control		Effect* or adjusted odds ratio (95% CI)	P value	Coefficient of variation
	No of participants	Mean* or proportion		No of participants	Mean* or proportion			
No of partners in past year:								
12 months	558	1.32		546	1.32	0.0001 (−0.0024 to 0.0049)	0.74	0.18
24 months	536	1.19		547	1.19	0.0001 (−0.0012 to 0.0025)	0.73	0.12
Any transactional sex with a casual partner:								
12 months	559	0.072		550	0.031	2.55 (1.19 to 5.46)	0.016	1.52
24 months	537	0.020		547	0.022	0.94 (0.41 to 2.18)	0.89	1.62
>1 incident of physical or sexual intimate partner violence:								
12 months	559	0.184		550	0.207	0.87 (0.64 to 1.18)	0.36	0.55
24 months	537	0.147		547	0.135	1.14 (0.77 to 1.68)	0.51	0.72
Pregnancy:								
12 months	537	0.145		518	0.129	1.14 (0.78 to 1.67)	0.49	0.70
24 months	521	0.144		534	0.116	1.45 (0.92 to 2.28)	0.11	0.82
Any casual partner:								
12 months	553	0.228		544	0.204	1.14 (0.82 to 1.59)	0.42	0.61
24 months	534	0.183		547	0.163	1.17 (0.85 to 1.63)	0.34	0.45
Correct condom use at last sex:								
12 months	533	0.552		516	0.558	0.96 (0.72 to 1.28)	0.79	0.29
24 months	520	0.575		534	0.596	0.90 (0.70 to 1.17)	0.45	0.24
Depression:								
12 months	559	0.154		550	0.122	1.32 (0.92 to 1.89)	0.13	0.69
24 months	537	0.128		547	0.161	0.76 (0.51 to 1.15)	0.20	0.74
Problem drinking:								
12 months	559	0.041		550	0.042	0.94 (0.45 to 1.95)	0.87	1.47
24 months	537	0.034		547	0.022	1.40 (0.61 to 3.17)	0.43	1.80
Ever misused drugs:								
12 months	530	0.024		513	0.039	0.60 (0.29 to 1.28)	0.19	1.17
24 months	437	0.023		423	0.019	1.20 (0.51 to 2.83)	0.68	1.29

*For No of partners in past year only.

Some of the other outcomes for men did show differences in the hypothesised direction (table 5). A significantly lower proportion of men in the intervention arm reported having had transactional sex with a casual partner at 12 months, although this difference had disappeared by 24 months. The proportion of men who perpetrated physical or sexual intimate partner violence was significantly lower in the Stepping Stones arm at 24 months, and there was some evidence that it was also lower at 12 months. There was some evidence that a lower proportion of men in the Stepping Stones reported raping or attempting rape at 12 months and that a lower proportion had any casual partner at 12 months. A significantly lower proportion of men in the Stepping Stones arm reported problem drinking at 12 months, and there was some evidence that a lower proportion were depressed at 24 months and that a lower proportion initiated drug misuse between 12 and 24 months.

**Table 5 tbl5:** Results for men: other outcomes at 12 and 24 months

	Stepping Stones			Control		Effect* or adjusted odds ratio (95% CI)	P value	Coefficient of variation
	No of participants	Mean* or proportion		No of participants	Mean* or proportion			
No of partners in past year:								
12 months	531	2.28		500	2.51	−0.0078 (−0.033 to 0.0001)	0.063	0.24
24 months	501	2.15		474	2.39	−0.0045 (−0.023 to 0.0003)	0.12	0.25
Any transactional sex with a casual partner:								
12 months	534	0.036		505	0.075	0.39 (0.17 to 0.92)	0.031	1.72
24 months	504	0.018		479	0.019	1.02 (0.39 to 2.65)	0.97	2.09
>1 incident of physical or sexual intimate partner violence:								
12 months	534	0.114		505	0.149	0.73 (0.50 to 1.06 )	0.099	0.63
24 months	504	0.062		479	0.096	0.62 (0.38 to 1.01)	0.054	0.67
Rape or attempted rape:								
12 months	534	0.092		505	0.123	0.71 (0.47 to 1.06)	0.094	1.86
24 months	501	0.080		473	0.085	0.92 (0.53 to 1.58)	0.76	1.91
Impregnated any woman:								
12 months	534	0.086		505	0.083	1.03 (0.66 to 1.62)	0.89	0.82
24 months	504	0.113		479	0.129	0.88 (0.60 to 1.31)	0.53	0.76
Correct condom use at last sex:								
12 months	513	0.735		483	0.689	1.26 (0.92 to 1.74)	0.16	0.22
24 months	485	0.732		462	0.751	0.88 (0.64 to 1.21)	0.43	0.20
Any casual partner:								
12 months	530	0.549		499	0.601	0.79 (0.59 to 1.05)	0.098	0.28
24 months	501	0.531		473	0.569	0.85 (0.62 to 1.15)	0.29	0.33
Depression:								
12 months	534	0.022		505	0.046	0.45 (0.16 to 1.21)	0.11	2.05
24 months	504	0.028		479	0.050	0.52 (0.24 to 1.13)	0.097	1.49
Problem drinking:								
12 months	534	0.198		505	0.265	0.68 (0.49 to 0.94)	0.021	0.57
24 months	504	0.266		479	0.257	1.10 (0.81 to 1.49)	0.56	0.52
Ever misused drugs:								
12 months	337	0.16		310	0.16	1.07 (0.65 to 1.77)	0.78	0.49
24 months	232	0.065		215	0.12	0.50 (0.23 to 1.11)	0.088	0.47

*For No of partners in past year only.

The aggregated cluster level analyses produced estimates and confidence intervals that were similar to those from the individual level analyses (results not shown). The per protocol analysis produced estimates that were similar to the intention to treat analysis, so the results are not shown.

## Discussion

Participation in the Stepping Stones programme in South Africa did not reduce the incidence of HIV infection among young men and women aged 15/26 but was associated with a reduced incidence of herpes simplex type 2 (HSV-2). There was no evidence of any desired behaviour change in women. There was more transactional sex with a casual partner at 12 months (but not at 24 months) among women in the Stepping Stones arm, and there was a suggestion of more unwanted pregnancies at 24 months. Men in Stepping Stones reported less transactional sex at 12 months, less perpetration of intimate partner violence (significant at 24 months, suggested at 12 months), less problem drinking at 12 months, and less drug misuse at 24 months. There was a suggestion of change in several other outcomes in men, including fewer partners at 12 months, less likelihood of casual partners, less rape at 12 months, and less depression at 24 months.

### Strengths

This was a randomised controlled trial had two biological outcomes. Few randomised controlled trials in Africa have evaluated behavioural interventions with biological outcomes and none has found clear evidence of effect. Our finding of an impact on HSV-2 infection (although not on HIV) is important for HIV prevention as Stepping Stones is a widely used intervention and HSV-2 is an important cofactor in heterosexual transmission of HIV. Meta-analysis indicates that people infected with HSV-2 have three times the risk of HIV infection.^19^

The impact of the intervention on incident infections of HSV-2 in women suggests that desirable behaviour change occurred in at least some women. A possible explanation for the lack of demonstrated impact on women’s behavioural outcomes is differential reporting bias—that is, under-reporting of sexual activity at baseline—with those who went through Stepping Stones becoming more forthright. This is a recognised problem with self reported behavioural outcomes.^5^ Alternatively Stepping Stones might have influenced unmeasured behaviour changes or choices of partners that protected against HSV-2 in previously unexposed women. Other authors have reflected that the ability of women to change their sexual behaviour in the context of unequal gender power relations is less than that of men.^5^ Young women are particularly at risk of being infected with HIV by older men (who have a higher age specific prevalence than younger men),^20^ and in these relationships the age differential further reduces women’s power. The prevalence of herpes is much higher in young men than that of HIV and so it is possible that some women were able to change their behaviour with younger male partners in a way that protected them from acquiring HSV-2 and this was somehow not reflected in the study’s behavioural outcomes. The findings from the qualitative research support this, as it was observed that women were sometimes able to change their behaviour with younger partners while not doing so with their older main partner. This raises the possibility of Stepping Stones having a positive longer term impact on women’s HIV risk beyond the period of observation of the study.

We observed changes in two other outcomes in women that were not in the intended direction. There was more transactional sex with a casual partner at 12 months among women in the Stepping Stones arm and there was a suggestion of more unwanted pregnancies at 24 months. Though the negative impact on transactional sex had resolved by 24 months, we suggest that particular care should be given to how transactional sex is discussed in groups of young women. Group discussions might have inadvertently encouraged transactional sex by reflecting it as at least common, if not standard, and an effective way of acquiring desired items. The attempts of facilitators to avoid being moralistic in discussions about transactional sex might have meant that the negative impacts were insufficiently emphasised.

The changes in sexual and violent behaviour of men were supported by the findings of qualitative research. Stepping Stones is a behavioural intervention that, according to a recent classification of interventions by WHO, is “gender transformative” in that it seeks to transform gender roles and promote more gender equitable relationships between men and women. 21

Our results suggest that it did lead to some change in violent and exploitative behaviour in men. Analyses performed on the baseline dataset showed that behaviours transformed by the intervention were those associated with perpetration of intimate partner violence,^22^ rape,^23^ and participation in transactional sex,^24^ and we hypothesise that these variables reflect particular ideas of masculinity. Evaluations of Stepping Stones in many other settings have documented an impact on men’s violence against their intimate partner,^11^ ^25^ which further supports our study’s findings. Stepping Stones is one of few interventions with demonstrated effectiveness in reducing this. The clearer visibility of this reduction at 24 months when compared with 12 months is consistent with the findings of other interventions^26^ and suggests that this positive behaviour change is being strengthened over time. Exposure to intimate partner violence has been identified as an important risk factor for HIV in women^27^ and so the reduction in male violence might have a broader impact on HIV in their sexual partners well beyond the study setting. Many of the other changes in men’s behaviour were not sustained to 24 months, which points to the need for research to strengthen the intervention.

### Weaknesses

The trial has several weaknesses that might affect the interpretation of the results. Randomisation occurred before recruitment. No villages declined to participate in the study because of their allocation but some individuals did. In both control and intervention clusters we usually had more volunteers for the study than we were able to include so it was not possible to count how many people dropped out during selection because of the allocation as opposed to other reasons, including the need to restrict recruitment to a maximum of 40 per cluster. We noted, however, that some people (particularly women) who lived far from the schools where the sessions were held thought they could not attend the whole Stepping Stones programme. Some women were not allowed to take part because they had strict parents who expected them home quickly after school. It is possible that those in the Stepping Stones arm were in some ways more motivated. This might have differentially influenced the response to the interventions. It is difficult to know how this would have affected our results and generalisability thereof, but given the fairly modest results of this trial, especially for women, it seems unlikely that there was a substantial impact.

The generalisability of the study findings could be influenced by several aspects of the trial design. The scope of our intervention was deliberately constrained by affordability in the design of the evaluation. We thus did not evaluate the model of programme delivery originally intended by Welbourn,^10^ which includes groups of older adult participants and multiple groups within the same village. Having done so might have enhanced the overall impact of the programme. This model reflects the socioecological perspective, which has been advocated in HIV prevention.^28^ We designed the trial to measure the impact of Stepping Stones when delivered in a way that reflected the practices of local organisations that work with such programmes. In so doing, our intention was to give some indication of the likely impact of Stepping Stones outside a trial setting. Any weaknesses in delivery of the intervention were probably no greater than those normally found. Our findings are a measure of the difference in outcomes between the two arms. For ethical reasons we provided a reasonably substantial control intervention that focused on HIV prevention and was taken from the Stepping Stones intervention. We cannot exclude the possibility that it resulted in behaviour change, although given the difficulties researchers face in showing impact from behavioural interventions^2^ ^3^ it would be surprising if the control intervention had a substantial impact.

The assumptions we used in calculating the required sample size for the trial were too optimistic. The effect size used in the sample size calculation was large (50% reduction in HIV incidence) and the anticipated overall incidence of HIV was incorrect. In addition, although we used a larger value for the coefficient of variation between clusters than was used in the Mwanza trial, the value used (0.35) was in fact considerably smaller than the actual value of 1.02. Our stratification of the clusters did not help in reducing the variation in incidence rates between clusters, which shows the practical difficulties of stratifying on surrogate geographical variables. Sample size calculations in future evaluations of behavioural interventions should use a more modest estimate of expected effect size and realistic estimates of either the coefficient of variation (*k*) between clustersor the intracluster correlation. The choice of *k* was informed by an analysis of the results of the 1999 national antenatal seroprevalence survey for the Eastern Cape. We thought that this value of *k* might be optimistic as the clusters in the antenatal survey (being antenatal clinics) cover a larger geographical area than the clusters proposed for the Stepping Stones intervention. The final value of *k*, however, was a compromise. We though that if we used too large a value of *k* in our sample size calculations, this would discourage funders. One positive recommendation from this study, which has been supported by other evaluations of behavioural interventions, is that large sample sizes are required to assess the modest but important reductions in incidence of HIV that might result from behavioural interventions and that necessary funding should be provided.

In the control arm slightly more blood specimens were collected by dried blood spot. Although this method for HIV testing was optimised, previous research has suggested that it might be slightly less sensitive than when serum is used.^29^ The impact of any such loss of sensitivity would have been to underestimate the true incidence of HIV in the control arm.

There could have been contamination between arms, but serious contamination is unlikely as clusters were geographically separated and the total sample size was small compared with the overall population, so the likelihood of participants forming friendships with people from the other study arm was low. Despite considerable efforts to trace cohort members, about 15% failed to contribute any data to the biological outcomes and a quarter were untraceable at 24 months. Our follow-up rates compare favourably with those of similar trials—for example, Ross et al lost 27% to follow-up.^5^ As follow-up rates were similar in the intervention and control arms this is unlikely to have biased the results.

### Implications

The meaning of the study findings is determined by an assessment of whether this was a trial with negative or positive findings. Some would argue that Stepping Stones did not work because it failed to affect the incidence of HIV. Literature on evaluation of behavioural interventions, however, rarely disregards all other outcomes, and we have shown significant other effects. We analysed the other biological outcome, incidence of HSV-2, across both years and found a reduction in the intervention arm. Most of the changes suggested in other behaviours were not sustained to two years, as is commonly found with evaluations of behavioural interventions, and we endorse the view that for behaviour change to be meaningful it must be enduring.^28^ In contrast, the impact on perpetration of intimate partner violence seems to have been strengthened over the two years of follow-up. This is a pattern that is recognised in the behavioural science literature^30^; it results from people having had an opportunity over time to reflect on their behaviour or for the environment to reinforce behaviours. Both HSV-2 and intimate partner violence are established risk factors for HIV and so the observation that Stepping Stones had an effect is of some interest.

**What is already known on the topic**

HIV prevention studies have had mixed results in terms of their impact on sexual behaviour
Most studies have been conducted in Western countries; in sub-Saharan Africa no programme has been shown to reduce sexually transmitted infections

**What this study adds**

The Stepping Stones programme did not lower incidence of HIV but did reduce the incidence of herpes simplex type 2 virus and male perpetration of intimate partner violence
Stepping Stones can affect some risk factors for HIV in young men and women

## Notes

We thank the Planned Parenthood Association of South Africa Eastern Cape Branch, our partner in the study intervention; the National Institute for Communicable Diseases for quality control, testing, and storage of specimens; Nelisiwe Khuzwayo (community coordinator); Leslie Setheni, Veliswa Gobinduku, Busiswa Mketo, Yandisa Sikweyiya, Mthokozisi Madiya, Bongwekazi Rapiya, Sanele Mdlungu, Ayanda Mxekezo, Lungelo Mdekazi, Nocawe Mxinwa, Andiswa Njengele, Mvuyo Mayisela, Philiswa Bango, Nobapostile Malu, Lizo Tshona, Khanyisile Bakan, Linda Shute, Lindiwe Farlane, Siya Kave, Bantu Waka, Zoleka Mbange (field nurses and field workers); Bomkazi Mnombeli, Engela Gerber, Alta Hansen (data management, data entry, and secretarial support); Daniel Kayongo, UNITRA (advice on biological aspects of the study); and Chief Z S Mtirara and all the members of the community advisory board. Mary Koss advised on questionnaire design and aspects of the study implementation.

Contributors: RJ was the project leader throughout the trial; wrote the proposal, led on the study design, intervention adaptation, and questionnaires; managed the study; directly managed the project from September 2004-April 2006; and did much of the data management and led drafting of the paper. MN contributed to the design of the intervention and questionnaires, developed operating plans for the implementation of the trial, was the project manager from September 2002-August 2004, and contributed to interpretation of the data. JL was project statistician, responsible for statistical aspects of study design, data management, and data analysis. NJ contributed to the design of the intervention and questionnaires, implementation of the trial, and interpretation of the data. KD contributed to the design of the study, data management, interpretation of the findings, and drafting of the paper. AP designed the protocols related to HIV and HSV-2 testing and quality control of the biological side of the study, and supervised the laboratory tests and storage of specimens and interpretation of results. ND contributed to the management of the study and the interpretation of the data. All investigators contributed to writing this paper. JL is guarantor.

Ethical approval: University of Pretoria and University of Witwatersrand Ethics Committees.

Provenance and peer review: Not commissioned; externally peer reviewed.

## Notes

**Cite this as:** *BMJ* 2008;337:a506
